# Chloride channel-3 regulates sodium-iodide symporter expression and localization in the thyroids of mice on a high-iodide diet

**DOI:** 10.3389/fnut.2025.1537221

**Published:** 2025-03-21

**Authors:** Meisheng Yu, Zhiqin Deng, Ke Wang, Xiangzhong Zhang

**Affiliations:** ^1^Department of Hematology, The Third Affiliated Hospital of Sun Yat-sen University, Guangzhou, China; ^2^Hand and Foot Surgery Department, The First Hospital Affiliated to Shenzhen University, Shenzhen, China; ^3^Department of Otolaryngology, Head and Neck Surgery, The First Affiliated Hospital of Xi’an Medical University, Xi’an, China

**Keywords:** chloride channel 3, sodium-iodide symporter, reactive oxygen species, iodide excess, thyroid gland, iodide conditions, basolateral and lateral membranes

## Abstract

**Introduction:**

Certain chloride channels and H^+^/Cl^−^ antiporters, such as chloride channel 3 (ClC-3), are expressed at the apical pole of thyrocytes, facilitating iodide (I^−^) efflux. However, the relationship between ClC-3 and I^−^ uptake remains unclear. Additionally, whether ClC-3 and reactive oxygen species (ROS) regulate sodium-iodide symporter (NIS) expression and localization under excessive I^−^ conditions remain underexplored.

**Methods:**

The expression and localization of ClC-3 in wild-type (WT), ClC-3 overexpression (OE) and ClC-3 knockout (KO) were detected by Western blotting (WB), immunohistochemistry and immunofluorescence, respectively. The ^131^I uptake of the thyroid was measured by thyroid function instrument. The expression and localization of NIS in normal and high iodide diet were detected, respectively. The role of ROS in the regulation of NIS by ClC-3 was observed.

**Results:**

ClC-3 expressions in thyrocytes were primarily localized to the basolateral and lateral membranes, in both ClC-3 OE and WT mice groups under normal I^−^ conditions. I^−^ uptake was significantly higher in WT and ClC-3 OE mice than in the ClC-3 KO mice under normal I^−^ conditions. The ClC-3 OE group exhibited a higher number of thyroid follicles with elevated NIS expression in the basolateral and lateral membranes than the WT and KO groups. In the ClC-3 KO group, the NIS was predominantly localized in the cytoplasm. In the WT group, NIS fluorescence intensity at the basolateral and lateral membranes increased after 48 h of excessive iodide exposure compared to 24 h. In ClC-3 OE mice, NIS, initially localized intracellularly after 24 h of excessive iodide exposure, was almost fully reintegrated into the basolateral and lateral membranes after 48 h. In contrast, in ClC-3 KO mice, NIS remained primarily cytoplasmic, with no significant change between 24 h and 48 h of I^−^ excess. ROS fluorescence intensity was significantly higher in the ClC-3 OE group than those in the WT and KO groups after 24 h of I^−^ excess. Pre-inhibition of ROS showed no significant differences in NIS localization or expression among the three groups after 24 h of I^−^ excess.

**Discussion:**

These findings suggest that ClC-3 may regulate NIS function via ROS signaling under excessive iodide conditions.

## 1 Introduction

Iodide is essential for thyroid hormone synthesis (1–3). As a significant regulator of thyroid function, excessive iodide can result in various conditions, such as hyperthyroidism and hypothyroidism, depending on the physiological status of the thyroid gland (4).

Extensive evidence demonstrates that these channels facilitate Cl^−^ transport, with some accommodating I^−^ passage (5). ClC-3, a member of the ClC-3/ClC-4/ClC-5 subfamily of chloride transport proteins, localizes to the plasma or intracellular membranes (6, 7). In human thyroid Nthy-ori3-1 cells, the anion permeability sequence of 17β-estradiol-sensitive channels favors I^−^ over Cl^−^ (8). Previous studies have indicated that ClC-3 participates in I^−^ efflux at the apical pole of rat thyroid cells (9). A previous study demonstrates that ClC-3 is primarily localized to the lateral and basolateral membranes of the SD rat thyroid under both control conditions and in the NaI-6-h group (9). However, whether ClC-3 is involved in iodide uptake at the thyroid basolateral membrane remains unclear.

The Na^+^/I^−^ symporter (NIS), a key protein responsible for thyroid iodide uptake (1, 2), is an integral plasma membrane glycoprotein localized to the basolateral side of thyrocytes (10–14). Excess iodide treatment significantly inhibits NIS expression in PCCI3 cells within 24 h, with NIS activity beginning to recover after 48 h (15). CFTR has been shown to facilitate NIS-mediated iodide uptake in pig thyroid epithelial cells by modulating the Na^+^ current (16, 17). Additionally, CFTR contributes to both iodide efflux and uptake (17). Consequently, examining the relationship between ClC-3 and iodide uptake, particularly its interaction with NIS, is essential, considering that excess iodide is a key regulator of NIS function (15, 18, 19). Growing evidence indicates that NIS expression, function, and subcellular localization are regulated by ROS-dependent mechanisms during I^−^ excess (20). Therefore, this study aims to investigate the relationship between ROS, ClC-3, and NIS under both physiological and excess iodide conditions. These findings could offer novel therapeutic insights into the mechanisms underlying the onset and progression of thyroid diseases.

## 2 Materials and methods

### 2.1 Materials

NaI was procured from Sigma-Aldrich (cat. #409286; St. Louis, MO, USA). Cell culture media were purchased from Gibco (cat. #21700–075; Thermo Fisher Scientific, Waltham, MA, USA). Additionally, ^131^I-NaI was obtained from Xiai Pharmaceutical Co., Ltd. (Guangdong, China). Thyroid ^131^I uptake was measured using a thyroid function analyzer (Zhongke Zhongjia Science Instrument Co., Ltd., Anhui, China).

### 2.2 Animals

Wild-type (WT), ClC-3 overexpression (CIC-3 OE) and ClC-3 knockout (ClC-3 KO) male FVB mice (3 months old) were obtained from Cyagen Biosciences Inc. and generously provided by Professor Mao (Guangdong Pharmaceutical University) and Dr. Deng (First Affiliated Hospital of Shenzhen University), respectively. ClC-3 protein expression was analyzed following previously established methods (9). For *in vivo* experiments, the mice were assigned to one of two groups: the normal iodide diet group, receiving 1.5 μg/day of iodide in deionized water through intragastric administration, while the excess iodide diet group, receiving 150 μg/day of iodide in deionized water through the same administration method over the same period. These conditions were maintained for 24 h and 48 h, respectively (21). All animal experiments were approved by the Institutional Animal Care and Use Committee of Jinan University and conducted following the guidelines of the Declaration of Helsinki and national regulations. To confirm the involvement of oxidative stress, the antioxidant N-acetyl-L-cysteine (NAC), a known reactive oxygen species (ROS) inhibitor, was administered. Mice were injected intraperitoneally with a saline solution of NAC (100 mg/kg/day, Sigma-Aldrich) (22).

### 2.3 Cell culture

Immortalized rat thyroid cells (FRTL-5) were obtained from the American Type Culture Collection (ATCC). These cells were maintained in Coon’s modified F-12 M medium (Gibco, USA) supplemented with 5% calf serum and a six-hormone (6H) cocktail, including bovine thyroid-stimulating hormone (TSH; 1 mU/mL; Sigma-Aldrich, St. Louis, MO, USA), insulin (10 μg/mL; Sigma-Aldrich), hydrocortisone (0.36 ng/mL; Sigma-Aldrich), transferrin (5 μg/mL; Sigma-Aldrich), glycyl-L-histidyl-L-lysine acetate (2 ng/mL; Sigma-Aldrich), and somatostatin (10 ng/mL; Sigma-Aldrich) (23). The cells were cultured at 37°C in an atmosphere of 5% CO_2_ and 95% air, with the culture medium replaced every 3–4 days. Subculturing was performed every 7 days, and cells between passages 5 and 15 were used for experiments (24).

### 2.4 Lentiviral-mediated gene transduction

Lentiviral-mediated transduction with HBLV-r-ClC-3shRNA-mcherry-BSD was used to knock down ClC-3 expression in FRTL-5 cells. Fluorescence imaging was performed 72 h post-transduction to assess transduction efficiency. Subsequently, protein extraction was conducted, followed by Western blot analysis to examine the effects of CIC-3 knockdown on FRTL-5 cells.

### 2.5 Western blot analysis

Proteins were extracted from thyroid tissues using RIPA lysis buffer. The protein concentration was measured using the bicinchoninic acid (BCA) protein assay kit (Beyotime, Shanghai, China). Overall, 30 μg of extracted protein was loaded onto a 12% sodium dodecyl sulfate (SDS)-polyacrylamide gel for electrophoresis. Following electrophoretic separation, proteins were transferred onto polyvinylidene difluoride (PVDF) membranes and blocked for 2 h at room temperature (RT) using 5% nonfat dried milk in Tris-buffered saline containing 0.1% Tween 20 (TBST). Subsequently, the PVDF membranes were incubated overnight at 4°C with primary antibodies at the following dilutions: CIC-3 (1:1000; Cell Signaling Technology, Danvers, MA, USA), NIS (1:800; Proteintech, Wuhan, China), and GAPDH (1:1000; Proteintech). After primary antibody incubation, the membranes were incubated for 2 h at RT with horseradish peroxidase (HRP)-conjugated AffiniPure Goat Anti-Rabbit IgG (H + L) secondary antibodies (1:10000; Proteintech) in TBST. Excess antibodies were removed by washing the membranes three times with TBST. Protein bands were detected using a chemiluminescence imaging system. Band intensities were normalized to GAPDH as the loading control, before being quantified using ImageJ software.

### 2.6 Immunofluorescence staining

#### 2.6.1 FRTL-5 cell preparation

FRTL-5 cells were seeded onto poly-D-lysine-coated glass-bottom dishes and incubated for 72 h. The cells were fixed with 4% paraformaldehyde for 15 min at RT. Permeabilization was conducted using 0.5% Triton X-100 for 5 min, followed by three washes with phosphate-buffered saline (PBS). To reduce nonspecific binding, the cells were blocked with 10% sheep serum in PBS for 2 h. Subsequently, the cells were incubated overnight at 4°C with a rabbit anti-NIS primary antibody (1:100, 24,324-1-AP, Proteintech). They were incubated for 2 h at RT in the dark with a Cy3-labeled Goat Anti-Rabbit IgG (H + L) secondary antibody (1:200; Beyotime). The cells were washed three times with PBS, with each wash lasting 5 min. Nuclei were stained with Hoechst for 5 min, followed by three additional 5-min washes with PBS.

#### 2.6.2 Thyroid tissue sample preparation

The thyroid gland was immediately excised and fixed in 4% formaldehyde for 7 days. Post-fixation, the tissue was embedded in paraffin wax. Following deparaffinization, rehydration, and antigen retrieval, the sections were incubated overnight at 4°C with rabbit anti-NIS antibody (1:100; Proteintech) or rabbit anti-ClCN3 polyclonal antibody (1:100; Cell Signaling Technology, USA). Subsequently, the sections were incubated for 50 min with fluorescent secondary antibodies (1:200; Beyotime) and Hoechst 33258 (1:1000 in PBS). DAPI was applied to stain the nuclei for 5 min at RT. Finally, colocalization analysis was performed using Zeiss LSM 880 software (9).

### 2.7 Immunohistochemical analysis

Thyroid tissue sections from WT and KO mice were harvested and fixed in 4% paraformaldehyde. The sections were incubated in 3% H_2_O_2_ for 25 min to quench endogenous peroxidase activity. Subsequently, the sections were washed three times with PBS, each wash lasting 5 min. Blocking was performed with 3% bovine serum albumin (BSA) for 30 min at room temperature to prevent nonspecific binding. The sections were subsequently incubated overnight at 4°C with a primary antibody against ClC-3 (1:100; Cell Signaling Technology). After washing, the sections were incubated with an HRP-conjugated secondary antibody for 50 min at room temperature. Following another PBS wash, 3,3′-diaminobenzidine tetrahydrochloride (DAB; Sigma-Aldrich) was applied to visualize antigen localization. The thyroid sections were counterstained with Mayer’s hematoxylin, washed, and mounted. Finally, the stained sections were investigated under a Nikon Eclipse E100 light microscope (25).

### 2.8 Detection of reactive oxygen species in frozen tissue sections

Frozen tissue sections from the three groups were thawed at 25°C until moisture had evaporated. Subsequently, a histochemical pen was used to outline the thyroid tissue, followed by the application of a self-fluorescence quencher for approximately 5 min. The sections were then rinsed with water for 10 min. ROS dye solution (D7008; Sigma-Aldrich) was applied to the tissue and incubated at 37°C in the dark for 30 min. The slide was permeabilized and washed three times with PBS. An anti-fluorescence quenching reagent (Servicebio G1401, Wuhan, China) was applied to the slide. The CY3-positive channel appeared red, with an excitation wavelength of 510–560 nm and an emission wavelength of 590 nm. Images were obtained with fluorescence intensity analyzed using ImageJ.

### 2.9 ^131^I uptake assay

To evaluate thyroid iodine uptake, ^131^I uptake measurements were conducted in WT, ClC-3 OE, and ClC-3 KO mice, all maintained on a standard iodide diet. Prior to measurement, each group was intraperitoneally injected with 200 μL of saline containing 18.5 MBq of ^131^I, administered 2 h before the assessment. Subsequently, the mice were euthanized *via* cervical dislocation. For thyroid function analysis, a thyroid function analyzer (USTC Zonkia Scientific Instruments Co. Ltd., Anhui Province, China) was used by positioning the thyroid glands at a fixed distance. The percentage of ^131^I uptake in the thyroid was calculated using the formula (26):


$$\begin{array}{c} \mathrm{percentage of}\;I131\\ \mathrm{uptake in the thyroid} \end{array}=\frac{\begin{array}{l} \mathrm{thyroid counts}-\\ \mathrm{background counts} \end{array}}{\begin{array}{l} \mathrm{counts of injected}\;\\ I131\;\mathrm{dose} \end{array}}x\;100$$

### 2.10 Statistical analysis

Data were presented as mean values with their respective standard errors (SEs). Statistical analysis was performed using GraphPad Prism 7 software (San Diego, CA, USA). A *p*-value below 0.05 was considered statistically significant.

## 3 Results

### 3.1 Reduced iodide uptake in the thyroid of ClC-3 knockout mice in a normal iodide diet

**Figure 1:**
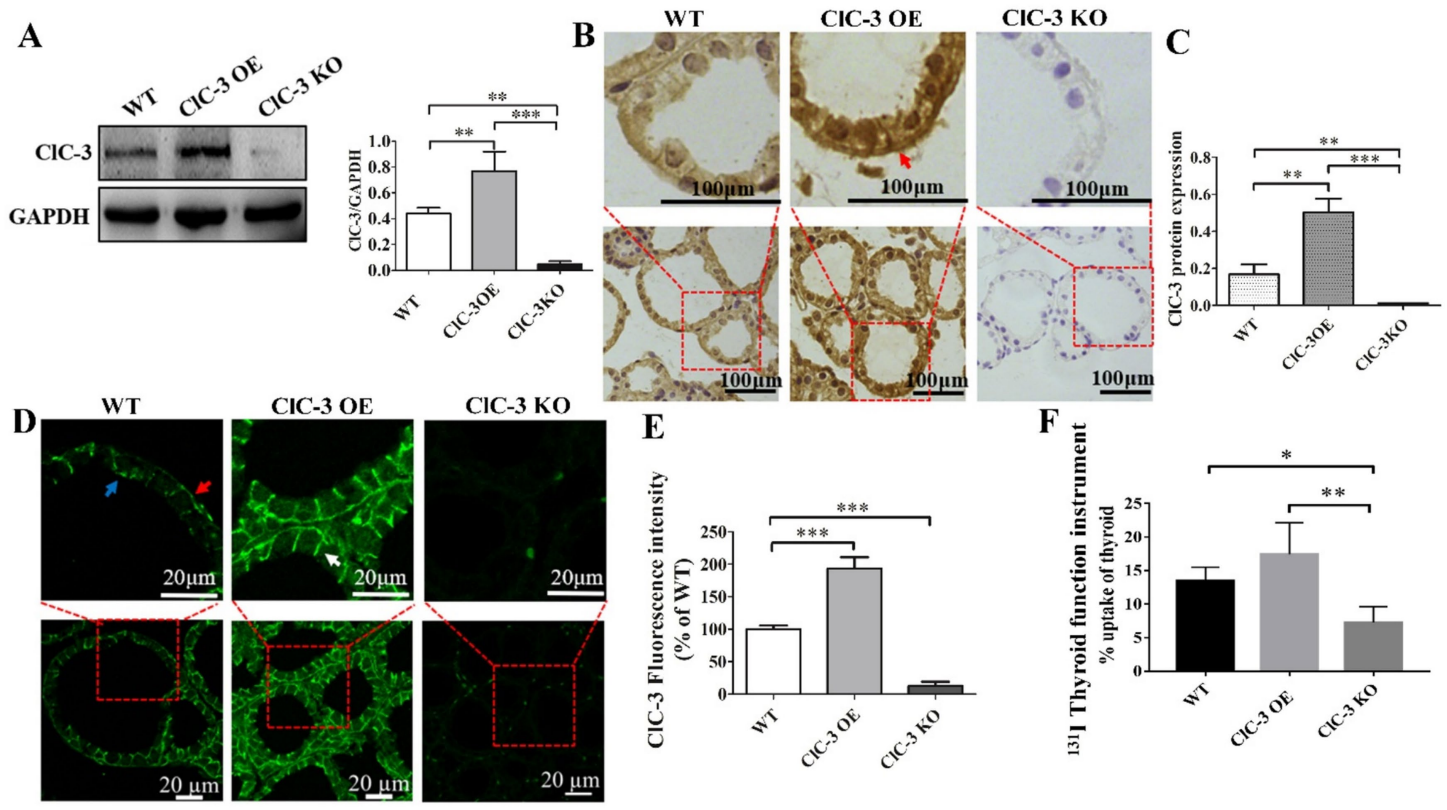
ClC-3 expression influences ^131^I uptake in the thyroid. **(A)** ClC-3 and control (GAPDH) protein expressions detected via Western blotting in WT, ClC-3 OE, or ClC-3 KO mice. Representative western blot and histograms of densitometric analyses normalized for the relative GAPDH content. **(B,C)** Immunohistochemical analysis of ClC-3 expression and location in the thyroid tissues of mice in the three groups. The brown color indicates specific immunostaining of ClC-3. The positive signal was measured via ImageJ. Original magnification: 400× **(B)**. **(D,E)** Thyroid sections from WT, ClC-3 OE, or ClC-3 KO mice fed a normal iodide diet were incubated with anti-ClC3 antibodies. Expression levels and distribution of ClC-3 were measured by immunofluorescence. Histogram analysis of the mean fluorescence intensity of ClC-3. **(F)** The percentage of ^131^I uptake in the thyroid was measured using a thyroid function instrument for WT, ClC-3 OE, and ClC-3 KO mice. Red arrows indicate ClC-3 staining on the basolateral side of the thyroid follicle, white arrows indicate ClC-3 staining on the lateral side, and blue arrows indicate ClC-3 staining on the apical side. All results are representative of three independent experiments. Data are presented as means ± SD (*n* = 6 for each group). *p < 0.05; **p < 0.01; ***p < 0.001. OE, overexpressing; KO, knockout; WT, wild-type.

Western blot analysis was conducted to evaluate ClC-3 protein expression across three groups of mice. ClC-3 expression was highest in the OE group, lower in the WT group, and lowest in the KO group (Figure 1A). Immunohistochemical analysis (Figures 1B,C) confirmed these findings, revealing a similar expression pattern to the Western blot analysis (Figure 1A). Immunofluorescence images revealed ClC-3 expression and localization in WT, OE, and KO mice (Figures 1D,E). In WT thyroid glands, ClC-3 was predominantly localized to the basolateral and lateral membranes of thyrocytes, with minimal expression at the apical pole. ClC-3 OE mice exhibited stronger ClC-3 fluorescence (labeled with Alexa Fluor 488, green) at the basolateral and lateral membranes than the WT mice (Figure 1D). ClC-3 KO mice exhibited no detectable fluorescence, indicating the absence of ClC-3 protein expression. ^131^I uptake was assessed in WT, ClC-3 OE, and ClC-3 KO mice, each receiving an intraperitoneal injection of 500 μCi Na[^131^I]. Thyroid radioiodide uptake was evaluated after 2 h using a thyroid function analyzer. The percentages of ^131^I uptake in WT, ClC-3 OE, and ClC-3 KO were 13.5 ± 2.04%, 17.4 ± 4.7%, and 7.3 ± 2.4%, respectively (Figure 1F).

### 3.2 Chloride channel 3 regulates sodium-iodide symporter expression and localization in the thyroid gland under a normal iodide diet

**Figure 2:**
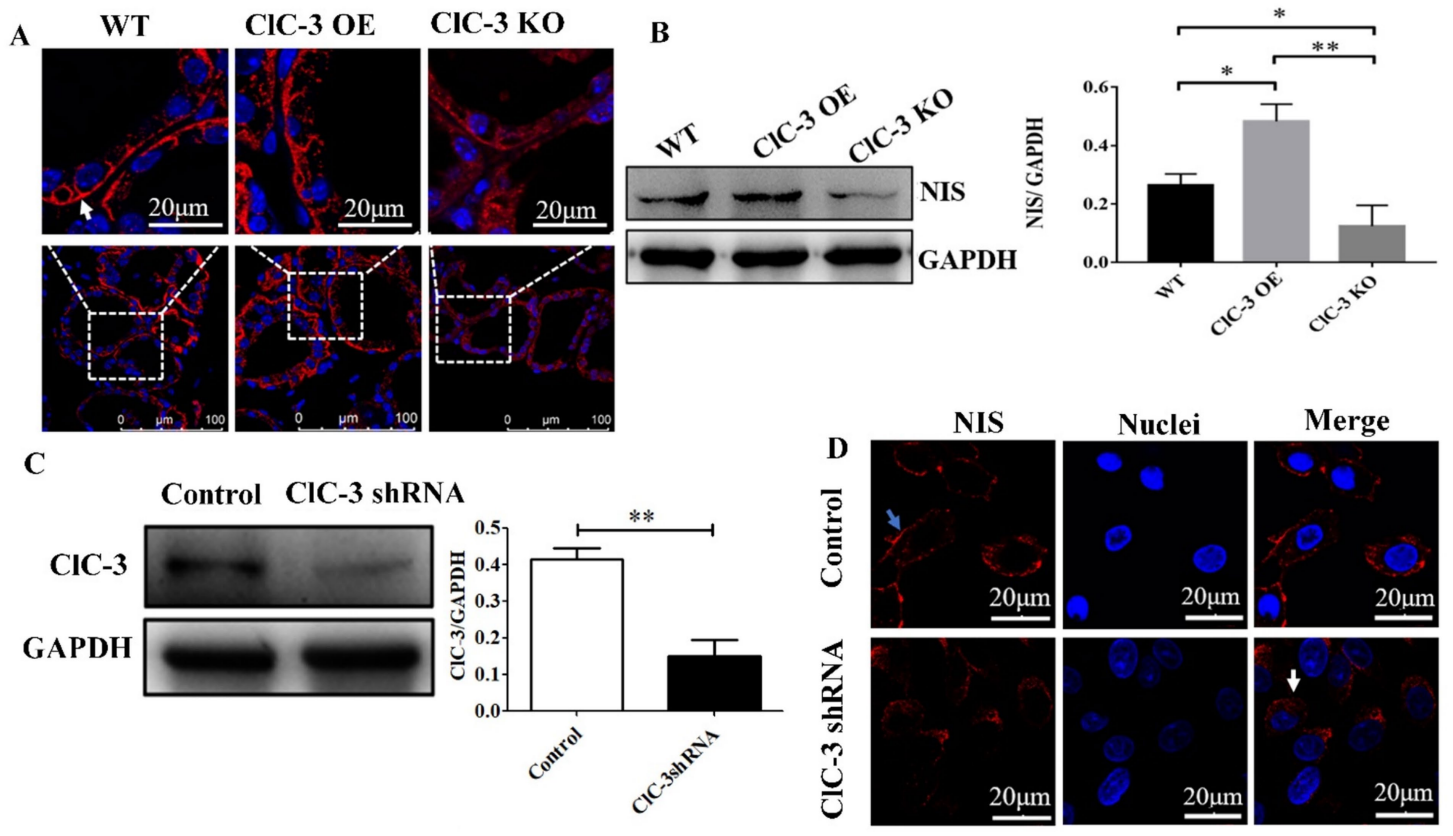
NIS protein expression or localization in the thyroid glands of WT, ClC-3 OE, and ClC-3 KO mice fed with normal iodide. **(A)** Immunofluorescence analysis was performed using a rabbit polyclonal anti-NIS antibody and an anti-rabbit fluorescein-conjugated secondary antibody, Cy3 (red). Nuclei were stained with DAPI (blue). The antibody predominantly labeled the basolateral membrane in the follicles (indicated by white arrows). Thyroid slides were observed under a Zeiss LSM 880 confocal microscope with a 63× immersion lens. **(B)** NIS proteins from the thyroid glands were extracted for western blot analysis (*n* = 6 per group). **(C)** ClC-3 and GAPDH proteins detected by Western blotting 72 h after shRNA transfection. Quantified expression levels of ClC-3 relative to GAPDH. **(D)** NIS expression and distribution in the control and ClC-3 shRNA of FRTL-5 cell lines. Blue arrows point to the cytomembrane, and white arrows point to the cytoplasm. Representative data from three experiments are displayed, with at least six animals per group used in each experiment. Data are presented as mean ± SD. *p < 0.05, **p < 0.01. OE, overexpressing; KO, knockout; WT, wild-type; NIS, Na^+^/I^−^ symporter.

Immunofluorescence analyses were conducted to evaluate the subcellular localization of NIS (labeled with Cy3, red) in thyrocytes, revealing its localization in the thyroid glands of WT, ClC-3 OE, and ClC-3 KO mice. In WT mice, NIS exhibited a characteristic linear staining pattern along the basolateral and lateral membranes of thyrocytes, similar to that observed in the ClC-3 OE group (Figure 2A). The ClC-3 OE group exhibited more thyroid follicles with high NIS expression at the basal membrane than the WT group. Furthermore, in the ClC-3 OE group, NIS was detected in the cytoplasm, compared to the WT group (Figure 2A). In the ClC-3 KO group, NIS expression at the basolateral membrane of thyroid follicles was generally lower, with a significant increase in cytoplasmic localization (Figure 2A). In ClC-3 KO mice, NIS protein levels in the thyroid glands were lower than in WT and ClC-3 OE mice, while the ClC-3 OE group exhibited higher NIS protein levels than the WT group (Figure 2B). In thyroid FRTL-5 cells, ClC-3 protein expression was significantly knocked down following treatment with ClC-3 shRNA for 72 h (Figure 2C). Further analysis of the FRTL-5 cell line revealed that in the ClC-3 shRNA group, NIS was predominantly localized in the cytoplasm, while in the control group, NIS was primarily distributed at the cell membrane under normal I^−^ conditions (Figure 2D). These findings suggest that ClC-3 affects both the localization and expression of NIS under normal iodide conditions (Figures 2A–D).

### 3.3 Excess iodide influences sodium iodide symporter localization and expression via ClC-3 regulation in the thyroid glands

**Figure 3:**
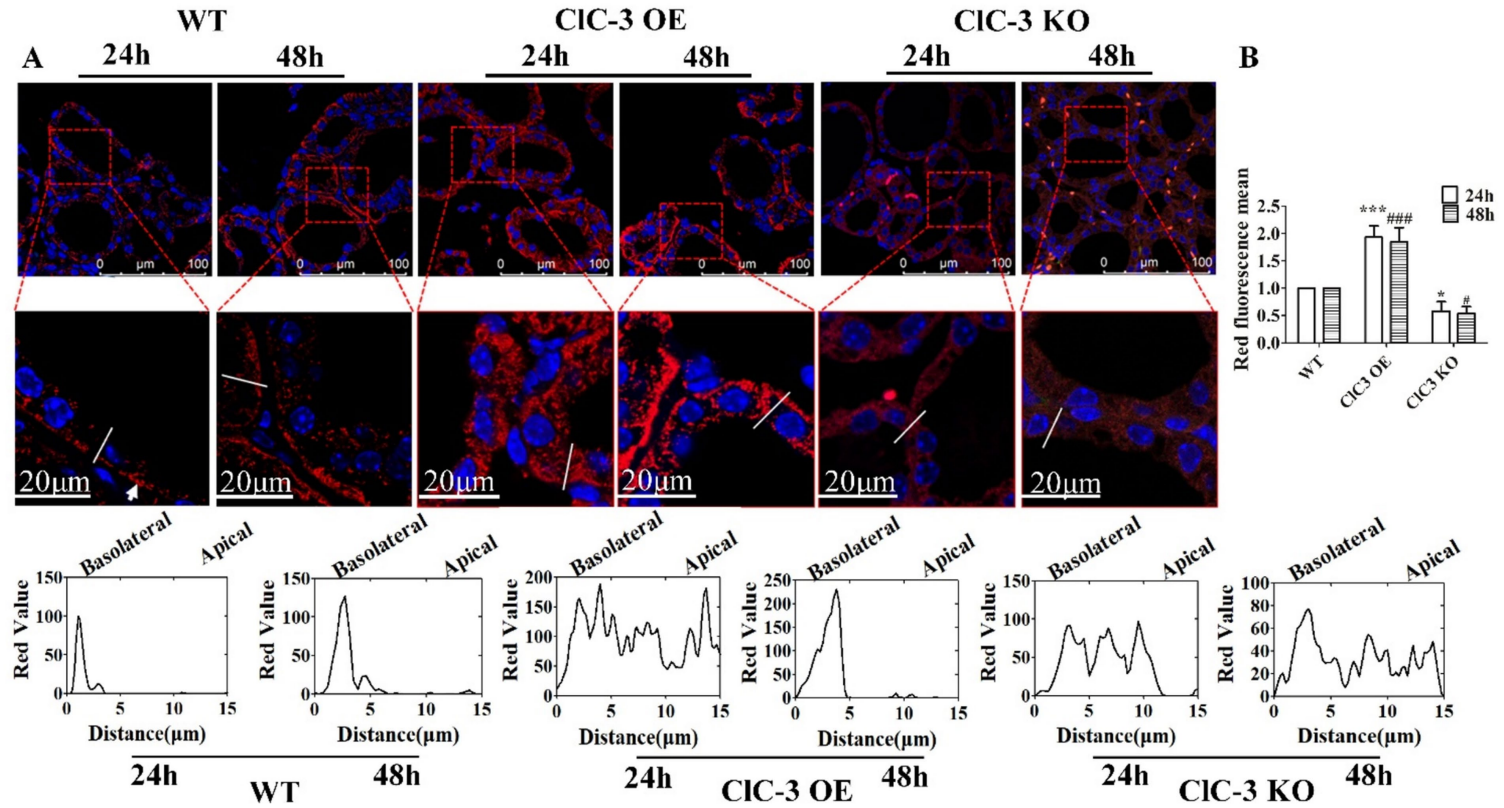
Immunofluorescence analysis of NIS expression and localization in WT, ClC-3 OE, and ClC-3 KO mice thyroid glands under excess I^−^. **(A)** Representative images from confocal immunofluorescence microscopy illustrate NIS expression and distribution in the thyroid follicles of WT, ClC-3 OE, and ClC-3 KO mice (*n* = 6 per group) after excess iodide administration (150 *μ*g/d NaI) for 24 or 48 h. The chart represents the analysis of NIS (red) fluorescence intensity across the white lines of 40 thyroid follicles in the three groups using ImageJ software. **(B)** Representative histogram images of total NIS fluorescence intensity detected by immunofluorescence after 24 h and 48 h under excess I^−^. NIS is visualized in red, and nuclei are visualized in blue. The white arrows represent the basolateral membrane of the thyroid cells. *p < 0.05, ***p < 0.001, compared with the WT (24 h), ^#^*p* < 0.05, ^###^*p* < 0.001, compared with the WT (48 h). Error bars represent the standard deviation. OE, overexpressing; KO, knockout; WT, wild-type; NIS, Na^+^/I^−^ symporter.

To further investigate the role of ClC-3 protein in the subcellular distribution of NIS in thyrocytes, immunofluorescence analyses were conducted on thyroid glands from WT, ClC-3 OE, and ClC-3 KO mice exposed to excess iodide for 24 h and 48 h. The normal iodide diet (1.5 *μ*g/day of iodide in deionized water) was replaced with an excess iodide diet (150 *μ*g/day of NaI in deionized water) for the respective durations before sacrifice. In the WT group, NIS protein distribution at the lateral and basolateral poles of thyrocytes increased after 48 h compared to after 24 h. In the ClC-3 OE group, NIS was predominantly localized intracellularly at 24 h but redistributed to the basolateral and lateral membranes of thyrocytes after 48 h. In contrast, in the ClC-3 KO group, NIS remained primarily cytoplasmic in thyrocytes at both 24 h and 48 h, with no significant changes in its localization or expression (Figure 3A). NIS total fluorescence intensity was higher in the thyroids of ClC-3 OE mice than in the WT and ClC-3 KO mice after 24 h and 48 h (Figure 3B). Cytoplasmic NIS fluorescence intensity was greater in ClC-3 OE mice than in the WT and ClC-3 KO mice after 24 h under excess I^−^ administration. These findings suggest that ClC-3 may influence NIS function during iodide overexposure.

### 3.4 Role of reactive oxygen species in ClC-3-mediated regulation of the sodium iodide symporter under excessive iodide conditions

**Figure 4:**
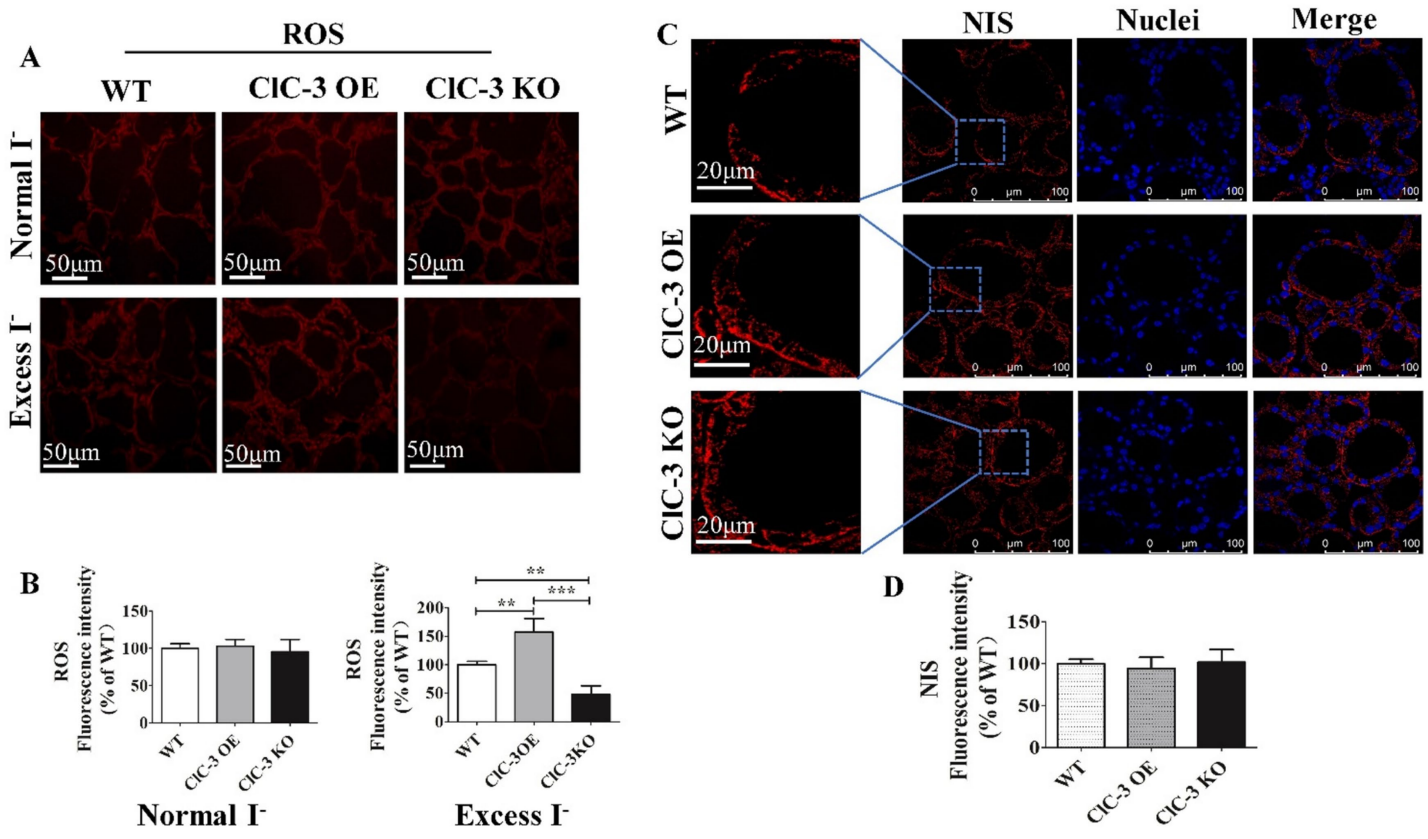
Effect of ROS on NIS expression and distribution in mice thyroid gland after 24 h under excess I^−^. **(A,B)** Red fluorescence represents the ROS intensity for frozen sections of the thyroid from three groups under normal I^−^ and excess I^−^ administration. **(C,D)** After inhibiting ROS production, representative images of NIS distribution and expression in WT, ClC-3 OE, and ClC-3 KO mice after inhibiting ROS production for 24 h under excess I^−^. The red fluorescence represents NIS, the blue fluorescence represents the nucleus, and the left column represents an enlarged view of a typical local region. Experiments were repeated three times with similar results. Data are presented as mean ± SD. **p < 0.01; ***p < 0.001. OE, overexpressing; KO, knockout; WT, wild-type; NIS, Na^+^/I^−^ symporter; ROS, reactive oxygen species.

As noted earlier, NIS expression and distribution varied significantly among the three groups 24 h after iodide excess. To further investigate the role of ROS in this process, we measured its fluorescence intensity, which was highest in ClC-3 OE mice, lower in WT mice, and lowest in ClC-3 KO mice. However, under normal iodide conditions, ROS levels in thyroid tissues did not differ significantly among the three mouse groups (Figures 4A,B). Pre-treatment with the antioxidant N-acetyl-L-cysteine (L-NAC) eliminated differences in NIS distribution and fluorescence intensity, resulting in a scattered cytoplasmic NIS pattern across all groups under excess iodide (Figures 4C,D). This suggests that ClC-3 regulation of NIS function under excess iodide conditions for 24 h may be ROS-dependent.

## 4 Discussion

Numerous studies indicate that the Cl^−^/H^+^ antiporter (ClC-5) and chloride ion channels, including CFTR and the calcium-activated chloride channel, are exclusively located in the apical membrane of thyrocytes, mediating I^−^ efflux (16, 27–32). Additionally, CFTR facilitates NIS-mediated I^−^ uptake in pig thyroid epithelial cells (17). ClC-3 is an intracellular transport protein primarily distributed in the late endosomal/lysosomal system, which can be transiently expressed in the plasma membrane before rapid endocytosis (7, 33, 34). Our findings indicate that ClC-3 is primarily expressed in the basolateral membrane of thyrocytes under normal I^−^ conditions, implying a potential role in iodide uptake. However, its relationship with the NIS function remains unreported. Emerging evidence suggests that ROS-dependent mechanisms regulate NIS expression and function under iodide overload (20). The pathway through which excess I^−^ induces ROS generation to affect NIS is still under investigation.

This study demonstrated that ClC-3 enhances ^131^I uptake in the thyroid with a normal iodide diet, prompting further investigation into its relationship with NIS function. In the ClC-3 KO group, NIS was significantly distributed in the cytoplasm, consistent with the lowest iodide uptake rate. This finding aligns with that of Mariano Martín et al. (13), who stated that NIS-mediated iodide uptake depends on its expression in the plasma membrane. However, Arriagada et al. reported that acute inhibition of iodide uptake due to excess iodide is mediated by increased ROS levels without altering NIS expression or localization in thyroid glands (18). This discrepancy may stem from variations in NIS distribution within the cytoplasm, influenced by cell type, iodide concentration, and observation time. To elucidate the regulatory pathway of ClC-3 on the role of NIS and ROS, NIS expression and localization were examined in WT, ClC-3 KO, and ClC-3 OE mice after 24 and 48 h of excess iodide intake. Excess iodide is a pivotal regulator of NIS function in the thyroid (18, 35). At high intracellular iodide concentrations, the Wolff-Chaikoff effect suppresses NIS protein expression by reducing its gene expression, inhibiting activity, and accelerating degradation. This effect diminishes after 2 days, restoring NIS activity and expression to normal (18, 35). Notably, in the ClC-3 OE group, NIS was translocated from the basolateral membrane to the cytoplasm after 24 h of excess I^−^ exposure. After 48 h under excess I^−^, most NIS proteins returned to the basolateral membranes, rapidly restoring NIS function and facilitating iodide uptake. Conversely, in ClC-3 KO mice, NIS predominantly remained in the cytoplasm after 24 and 48 h of excess iodide, suggesting that ClC-3 knockdown severely impairs NIS function and thyroid iodide uptake. These findings underscore the critical role of ClC-3 in thyroid self-regulation. Some studies indicate that thyroid cancers lose radioiodine avidity due to NIS repression and internalization from the basolateral membrane to the intracellular compartment (36–38). Therefore, the role of ClC-3 in thyroid diseases warrants further investigation.

Our study found that ClC-3 promotes ROS generation in an iodide-excess environment for 24 h. When ROS levels were suppressed by N-acetyl-L-cysteine, inhibiting ROS significantly impacted the regulation of NIS function by ClC-3. Despite the promising implications of our findings, the study has limitations. We have not studied the role of ROS at multiple time points in the regulation of NIS by ClC-3 under high-iodide diet. Future research should focus on exploring the mechanism by which ROS participates in the regulation of NIS in the cytoplasm of thyrocytes by ClC-3 under a high-iodide diet.

In conclusion, ClC-3 may enhance thyroid iodide uptake by influencing NIS expression and localization, with ROS potentially mediating this process under excess iodide. These findings suggest that ClC-3 is involved in thyroid autoregulation and provides insight into thyroid disease mechanisms.

## Data Availability

The original contributions presented in the study are included in the article/supplementary material, further inquiries can be directed to the corresponding authors.

## Glossary

WT: wild-type
CFTR: cystic fibrosis transmembrane conductance regulator
L-NAC: N-acetyl-L-cysteine
DAB: 3,3′-diaminobenzidine tetrahydrochloride
BSA: bovine serum albumin
PBS: phosphate-buffered saline
HRP: horseradish peroxidase
TBST: Tris-buffered saline containing 0.1% Tween 20
RT: room temperature
PVDF: polyvinylidene difluoride
SDS: sodium dodecyl sulfate
BCA: bicinchoninic acid
TSH: thyroid-stimulating hormone
ATCC: American Type Culture Collection
6H: six-hormone
ClC-3 OE: ClC-3 overexpression
ClC-3 KO: ClC-3 knockout
NIS: Na+/I− symporter
ROS: reactive oxygen species
I^−^: iodide
ClC-3: chloride channel-3
